# Stable In Vitro Cultivation of the Myxozoan *Enteromyxum leei* for over 12 Months: Integration of a Physical Aggregation Technique for Long-Term Antiparasitic Interventions

**DOI:** 10.3390/pathogens15080836

**Published:** 2026-08-10

**Authors:** Eun Ji Jeon, Han Gill Seo, Na Young Kim, Ahran Kim, Myoung Sug Kim, Jeong Wan Do, Hyun Ja Han, Hyun Sil Kang, Soo Ji Woo

**Affiliations:** 1 Pathology Division, National Institute of Fisheries Science, Busan 46083, Republic of Korea; 2 Aquaculture Industry Research Division, South Sea Fisheries Research Institute, National Institute of Fisheries Science, Yeosu 59780, Republic of Korea; 3 Subtropical Fisheries Research Institute, National Institute of Fisheries Science, Jeju 63068, Republic of Korea

**Keywords:** emaciation disease, myxozoa, *Paralichthys olivaceus*, physical purification

## Abstract

Emaciation disease, primarily caused by the *myxosporean* parasite *Enteromyxum leei*, poses a significant economic threat to the olive flounder (*Paralichthys olivaceus*) aquaculture industry. Research on *E. leei* has been severely restricted by its status as an unculturable obligate parasite and its extreme sensitivity to cryopreservation. In this study, we developed a standardized in vitro culture system that achieved stable maintenance of *E. leei* for over 12 months for the first time. To overcome the challenges of primary isolation, a physical aggregation technique was implemented, which effectively concentrated the parasites while reducing bacterial contamination by 99.1%, as validated by qPCR. The established culture, maintained in minimum essential medium supplemented with 30% FBS at 25 °C, exhibited a consistent 20-day asexual proliferative cycle consisting of six distinct developmental stages. Morphological stability and genetic identity were rigorously monitored, showing no phenotypic drift or genetic divergence throughout the 12-month period. Our results demonstrate that the entire proliferative phase of *E. leei* can be recapitulated in vitro without host intervention. Given that cryopreservation remains non-viable, this continuous culture system provides a critical, unique platform for high-throughput drug screening and myxozoan developmental biology, offering a robust tool to mitigate emaciation syndrome in marine fish farms.

## 1. Introduction

The aquaculture of olive flounder (*Paralichthys olivaceus*) represents a vital sector of Republic of Korea’s marine industry, particularly in Jeju Island. However, sustainable production is severely hampered by emaciation disease, a chronic parasitic condition that leads to substantial economic losses through high morbidity and stunted growth [1,2,3,4]. The primary etiologic agents of this syndrome are the *myxosporean* parasites *Enteromyxum leei* and *Parvicapsula anisocaudata*, which target the digestive tract and kidneys, respectively [5,6]. *Enteromyxum leei* is a particularly devastating histophagous pathogen that invades the intestinal epithelia, causing severe malabsorption and progressive metabolic decline [7]. Clinically, infected fish exhibit distinct hallmarks such as sunken eyes, cranial protrusion, and a drastic reduction in body weight [8], often resulting in a condition factor (K-factor) below 70% [9].

Since its first report in Japanese tiger puffer (*Takifugu rubripes*) in 1990 [10], the host range of *E. leei* has expanded globally, affecting various high-value species in Republic of Korea, including olive flounder since 2005 [11] and starry flounder (*Platichthys stellatus*) more recently in 2023 [12]. In 2024, despite Jeju’s *P. olivaceus* production reaching 19,900 tons [13], emaciation disease has imposed an annual economic burden exceeding USD 13 million due to increased mortality and stunted growth. This ongoing epidemic severely undermines the industry’s productivity and sustainability.

Despite the global expansion of its host range and the profound impact on the industry, the development of effective preventive or therapeutic measures has been hindered by a critical bottleneck: the inability to culture the parasite in vitro. As an obligate parasite with a complex life cycle and high host dependency, *E. leei* is notoriously difficult to maintain outside a living host [14]. Previous studies have largely relied on in vivo experimental infections or short-term maintenance of isolated stages [15,16], which limits the potential for high-throughput drug screening. Furthermore, unlike many other common fish parasites, *E. leei* exhibits extreme sensitivity to cryopreservation, necessitating a continuous and stable in vitro cultivation system to ensure research continuity.

In this study, we report a significant technical breakthrough in the management of this pathogen. The aim of the present study was to develop a standardized protocol for high-purity isolation and, for the first time, achieve stable in vitro maintenance of *E. leei* for over 12 months. This established culture system preserves the morphological integrity and viability of the parasite without host intervention, providing an essential platform for investigating its life cycle and developing targeted antiparasitic interventions.

## 2. Materials and Methods

### 2.1. Ethical Statement and Fish Sampling

All experimental protocols were conducted in accordance with the institutional guidelines of the National Institute of Fisheries Science. A total of 15 *Paralichthys olivaceus* (35 ± 2 cm, 454.5 ± 21 g) exhibiting typical emaciation symptoms, such as cranial protrusion and sunken eyes, were collected from commercial land-based farms in Jeju, Republic of Korea. The collected fish were ethically euthanized by immersion in an overdose of Tricaine methanesulfonate (MS-222; Sigma-Aldrich, St. Louis, MO, USA) at 250 mg/L, followed by cervical dislocation to ensure rapid anesthesia and termination prior to tissue dissection.

### 2.2. Isolation of Enteromyxum leei

Infected fish were euthanized, and the posterior intestine and rectum identified as the primary infection sites were aseptically excised. The mucosal layer was gently scraped using a sterile scalpel to collect intestinal mucus. The scrapings were suspended in sterile PBS and examined using an inverted light microscope (Eclipse TS100, Nikon, Tokyo, Japan) at 400× magnification to confirm the presence of *E. leei* proliferative stages.

### 2.3. Establishment of In Vitro Culture and Purification

The collected mucus was suspended in Minimum Essential Medium (MEM, Welgene, Gyeongsan, Republic of Korea) supplemented with 10% Fetal Bovine Serum (FBS, Atals Biologicals, Fort Collins, CO, USA) and 5% antibiotic-antimycotic solution (Gibco, Grand Island, NY, USA). The suspension was distributed into 48-well plates and incubated at 20–25 °C. To achieve high-purity isolation, a stepwise expansion and purification process was performed. For stepwise expansion, the primary mucus isolates were initially incubated in 48-well plates for 7 days until a stable proliferative density (≥5 × 10^3^ cells/well) was achieved; the cultures were then transferred to 12-well plates for 14 days, and finally expanded into 6-well plates upon satisfying the criteria of a confluent trophozoite cluster density (≥2 × 10^4^ cells/well) paired with a Trypan blue viability of ≥90% to successfully increase the culture area. For physical purification, a physical aggregation technique was applied by swirling manually in a uniform circular motion with an approximate diameter of 20 mm for 1 min. This operation generated a controlled centripetal force that effectively concentrated the denser *E. leei* trophozoites into a distinct, centralized pellet, while lighter host debris and free bacteria remained suspended at the periphery. The concentrated parasites were then selectively collected using a sterile micropipette and transferred to fresh medium to eliminate contaminants such as host blood cells and bacteria.

### 2.4. Quantitative Evaluation of Purification Efficiency

To validate the efficacy of the physical aggregation method, genomic DNA (gDNA) was extracted at two distinct stages: pre-purification, representing the initial culture media collected immediately after intestinal mucus isolation, and post-purification, consisting of the final aggregated parasite pellets after the removal of host debris. Total genomic DNA from both samples was extracted using a DNeasy Blood & Tissue Kit (Qiagen, Hilden, Germany) according to the manufacturer’s instructions. Quantitative real-time PCR (qPCR) was performed using the CFX96 Touch Real-Time PCR Detection System (Bio-Rad, USA). Specific primers were used to target three genomic markers: *P. olivaceus β-actin* (host DNA indicator), bacterial *16S rRNA* (microbial contamination), and *E. leei 18S rRNA* (target parasite) (Table 1). The amplification protocol consisted of an initial denaturation at 95 °C for 30 s, followed by 40 cycles of denaturation at 95 °C for 10 s and annealing/extension at 60 °C for 15 s. A final extension step was carried out at 72 °C for 5 min. The relative abundance of host and bacterial DNA was normalized to *E. leei 18S rRNA* levels using the 2^ΔΔ^ CT method [17].

### 2.5. Long-Term Maintenance and Optimization

Cultures were maintained for 12 months with medium replacement every four days. Subculturing was performed monthly by centrifugation (3000 rpm for 3.5 min) and reseeding into new 6-well plates. To optimize growth, proliferation efficiency was evaluated across a temperature range (16 °C, 20 °C, and 25 °C) and FBS concentrations (10%, 20%, and 30%). Cultures were maintained for 12 months with medium replacement every four days. To ensure strict counting consistency and eliminate operator bias during long-term cultivation, all parasite enumerations were performed by a single investigator using a hemocytometer equipped with a digital camera overlay. A 10 μL aliquot of the parasite suspension was collected from each well after thorough but gentle pipetting to ensure uniform distribution. The total number of viable proliferative stages was counted in triplicate for each of the three independent biological replicates at each time point. Continuous microbial safety monitoring was conducted throughout the 12-month period; during every monthly subculturing step, 100 μL of culture supernatant was routinely plated onto Tryptic Soy Agar (TSA) and Sabouraud Dextrose Agar (SDA) to screen for cryptic bacterial and fungal contamination, respectively, ensuring the system remained consistently uncontaminated after the physical purification phase.

### 2.6. Viability and Morphological Stability Assessment

Parasite viability was monitored using the Trypan blue exclusion assay. A 10 μL aliquot of 0.4% Trypan blue (Sigma-Aldrich, St. Louis, MO, USA) was mixed with the parasite suspension (1:1 ratio). After 1 min of incubation, the samples were observed under an inverted microscope. Viable parasites with intact membranes excluded the dye, while dead cells exhibited blue cytoplasmic staining. Morphological stability was documented monthly by comparing in vitro stages with the initial isolates.

### 2.7. Molecular Identification and Phylogenetic Analysis

Genomic DNA was extracted from the cultured parasites, followed by PCR amplification using a protocol and primers modified from a previous study [19]. The *18S ribosomal RNA* gene (1468 bp) was amplified using the specific primers EL-F (5′-GAT GAA ACT GCG AAG CGC TC-3′) and EL-R (5′-CAC AAG TTG ATG ACT TGC GC-3′). The thermal cycling conditions consisted of initial denaturation at 95 °C for 3 min, followed by 37 cycles of 95 °C for 30 s, 55 °C for 30 s, and 72 °C for 45 s, with a final extension at 72 °C for 5 min. The resulting PCR products were resolved by agarose gel electrophoresis on a 1.5% (*w*/*v*) agarose gel containing ethidium bromide alternative nucleic acid stain (SafeView, CleanAct, Seoul, Republic of Korea) in 1× TAE buffer at 100 V for 30 min. DNA bands were visualized and documented under ultraviolet transillumination using a digital gel imaging and documentation system (GelDoc, Bio-Rad, Hercules, CA, USA). The amplicons were sequenced and aligned with reference sequences from NCBI GenBank using ClustalW. The *18S rRNA* sequence of the isolated *E. leei* Korea strain was officially deposited in the GenBank database under the accession number PZ447519. A phylogenetic tree was constructed using the Maximum Likelihood (ML) method within the MEGA12 (Mega Software, State College, PA, USA) software package to evaluate evolutionary relationships [20].

### 2.8. Statistical Analysis 

All quantitative data, including purification efficiency and proliferation kinetics, are expressed as mean ± standard deviation (SD) from three independent biological replicates (*n* = 3). Normal distribution and homogeneity of variance were verified using the Shapiro–Wilk test and Levene’s test, respectively. Statistical comparisons among groups were evaluated by one-way analysis of variance (ANOVA) followed by Tukey’s post-hoc multiple comparison test. All statistical analyses were performed using SPSS software (version 27.0; IBM Corp., Armonk, NY, USA), and differences were considered statistically significant at *p* < 0.05.

## 3. Results

### 3.1. Clinical Characteristics and Isolation of Enteromyxum leei

**Figure 1:**
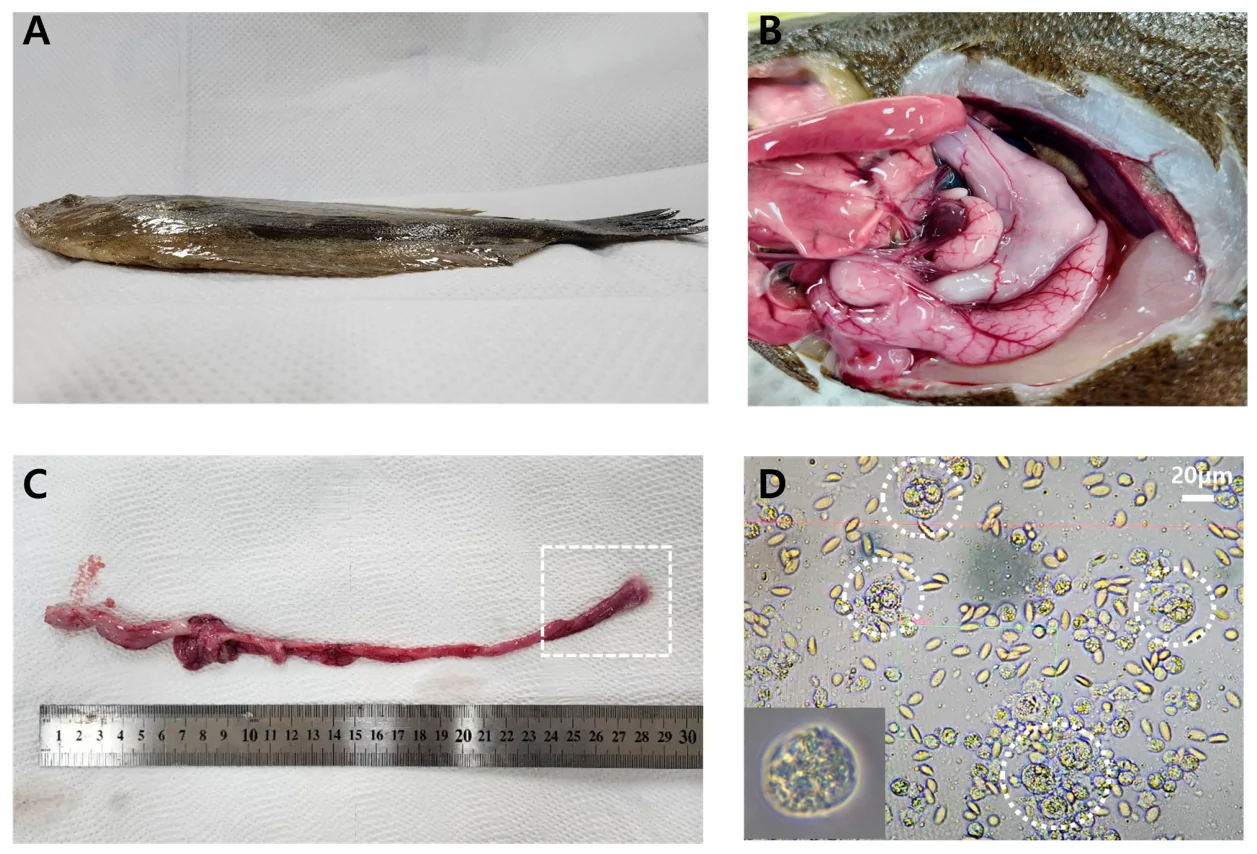
Clinical signs of emaciation disease in *Paralichthys olivaceus* and isolation of *Enteromyxum leei*. (**A**) Infected fish displaying typical emaciation symptoms, including severe wasting and cranial protrusion. (**B**) Clinical signs of an infected fish showing intestinal congestion, kidney enlargement, and hepatic congestion. (**C**) Excised intestinal tract; the dashed box indicates the posterior intestine and rectum targeted for isolation. (**D**) Light micrograph (400×) of intestinal mucus showing high-density *E. leei* proliferative stages (circles).

Infected *P. olivaceus* exhibited hallmark clinical signs of emaciation disease, most notably severe muscle wasting and prominent cranial protrusion (Figure 1A,B). Gross pathology revealed a translucent, thinned intestinal wall with yellowish mucoid contents. Microscopic analysis of mucosal scrapings revealed a high density of *E. leei* trophozoites embedded within host epithelial debris (Figure 1C,D).

### 3.2. High-Purity Culture via Physical Aggregation

**Figure 2:**
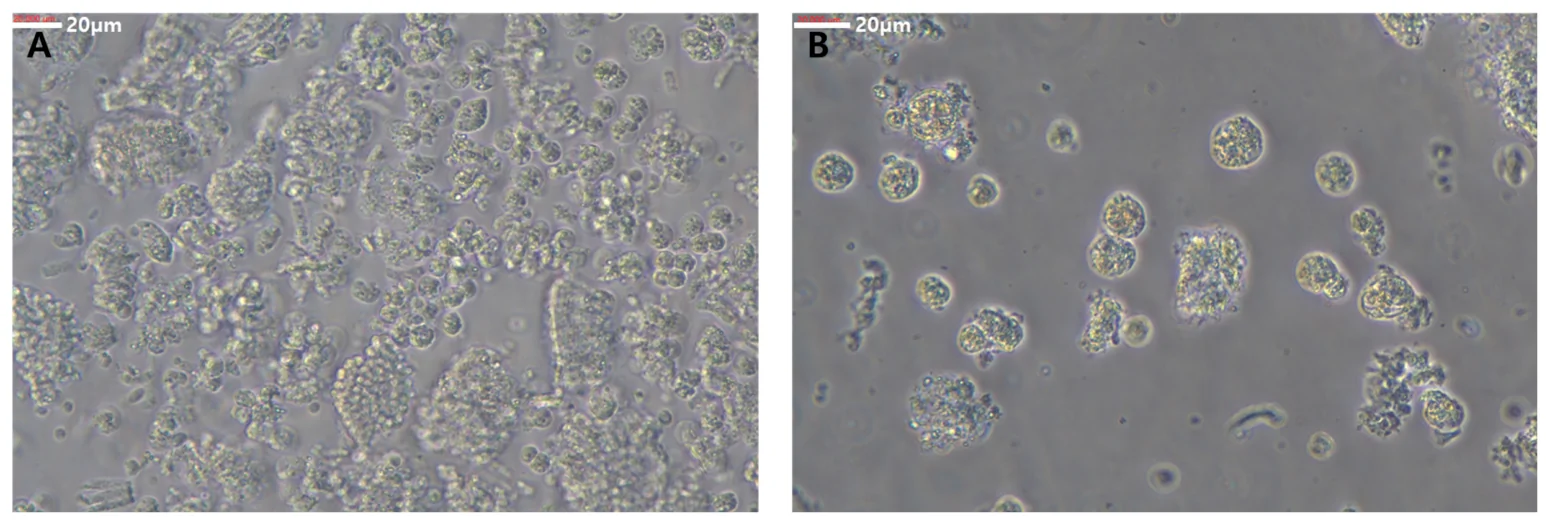
Enhancement of *E. leei* purity through the physical aggregation technique. (**A**) Pre-purification: Initial suspension containing host blood cells and epithelial debris. (**B**) Post-purification: High-purity culture following physical aggregation, showing effective elimination of contaminants. Scale bar = 20 μm.

**Figure 3:**
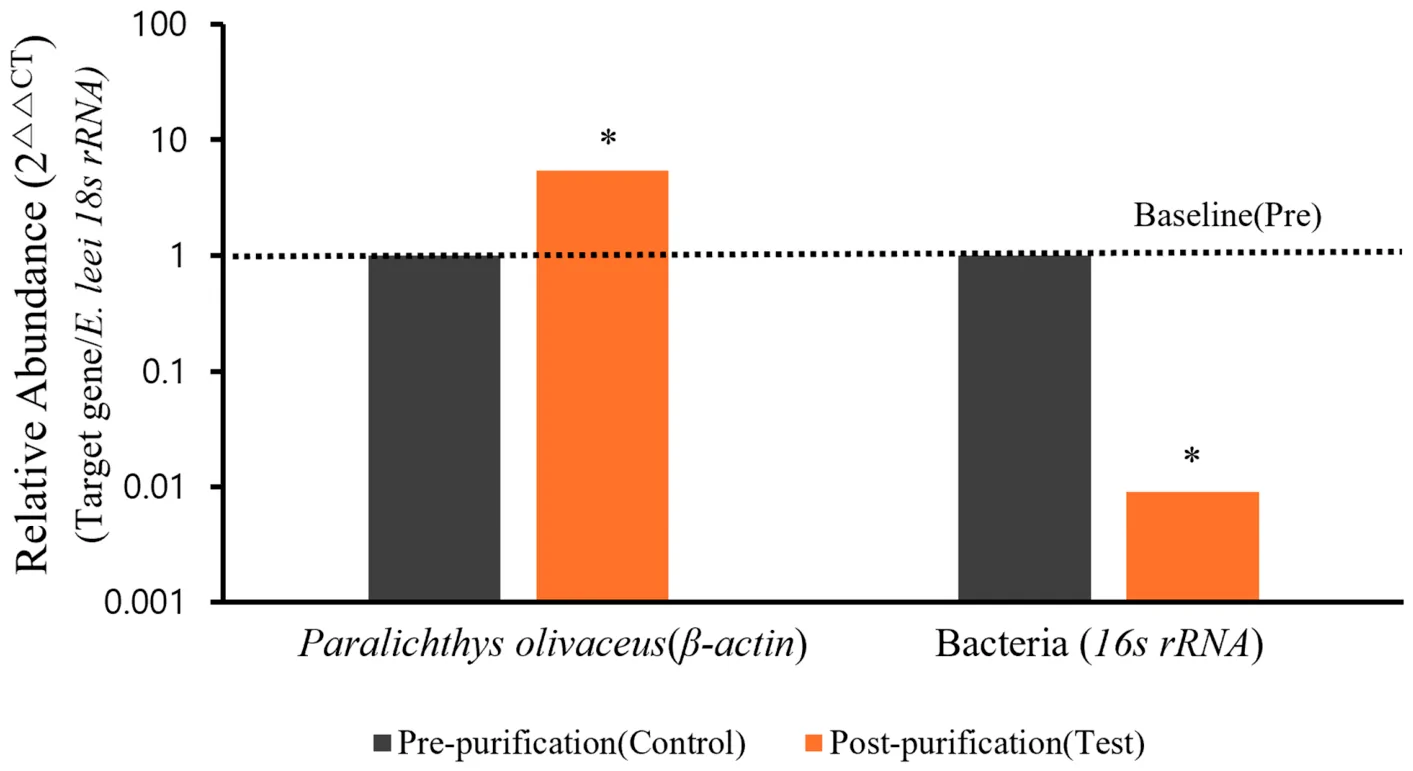
Quantitative validation of *E. leei* purification efficiency via qPCR. The relative genomic abundance of host-derived DNA (*β-actin*) and bacterial DNA (*16S rRNA*) was evaluated before and after physical aggregation. All values were normalized to *E. leei 18S rRNA*, with the pre-purification level set as the baseline (1.0). Statistical differences were obtained using one-way ANOVA followed by Tukey’s multiple comparison test with * *p* < 0.05. Data are presented as mean ± SD (*n* = 3).

Initial isolates were heavily contaminated with host-derived materials, including erythrocytes and sloughed epithelial cells, which hindered long-term maintenance (Figure 2A). By implementing the physical aggregation technique using controlled circular agitation, we successfully concentrated *E. leei* trophozoites into a centralized pellet while centrifugal forces moved lighter host debris to the periphery. This resulted in a significantly clarified culture field with a high enrichment of viable parasites (Figure 2B). Quantitative purity enhancement was validated by qPCR analysis showing a significant 99.1% reduction (0.009-fold) in bacterial *16S rRNA* levels (Figure 3). The relative increase in host DNA (5.41-fold) suggests the co-aggregation of microscopic host debris with parasite clusters. Genomic quantification data provided in Table S1.

### 3.3. Morphological Stability During 12-Month Continuous Cultivation

**Figure 4:**
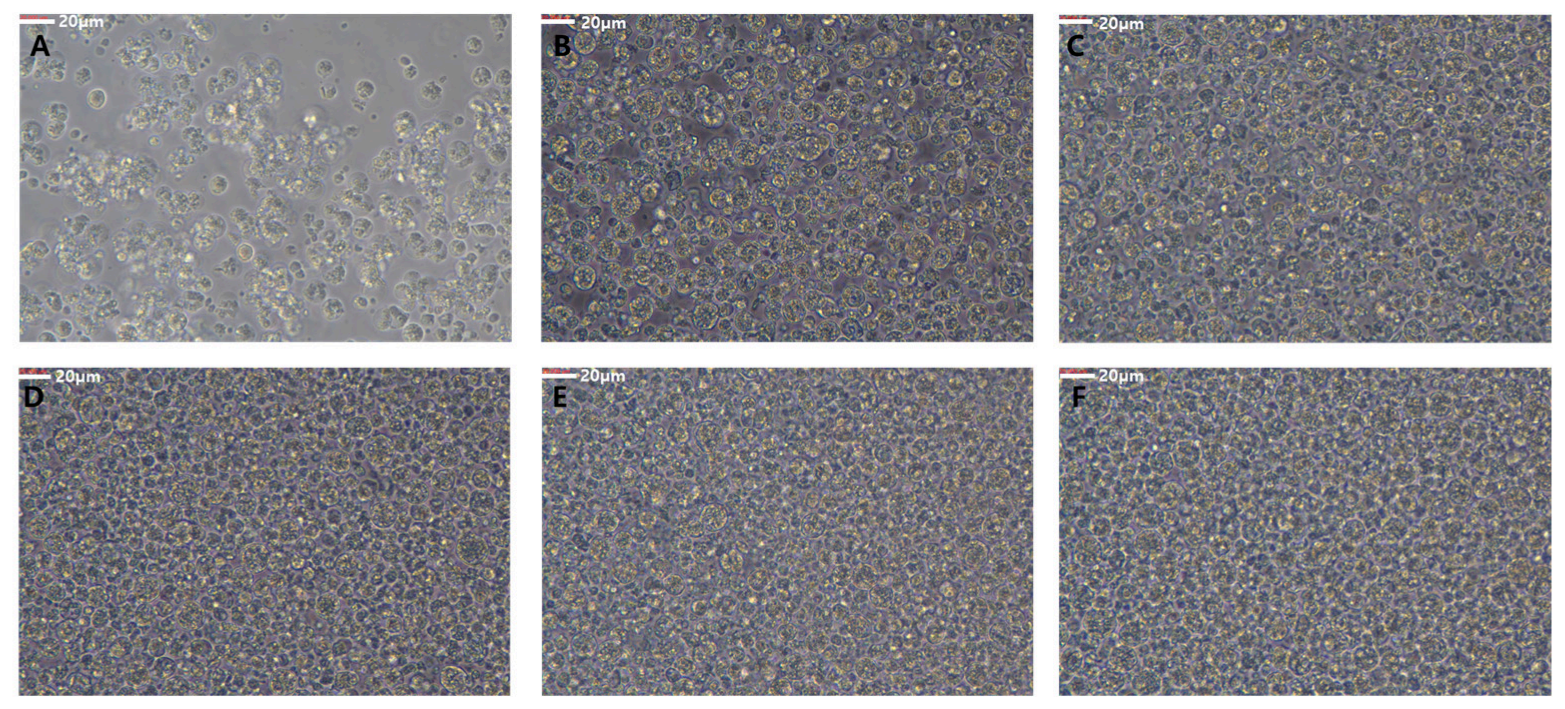
Morphological stability of *E. leei* during 12 months of continuous in vitro cultivation. (**A**) Immediately after isolation from the intestinal mucus of *P. olivaceus*. (**B**–**F**) Parasites maintained through continuous subculturing at (**B**) 1 month, (**C**) 2 months, (**D**) 3 months, (**E**) 6 months, and (**F**) 12 months post-isolation. Scale bar = 20 μm.

*E. leei* maintained consistent morphological characteristics throughout the 12-month culture period. The parasites retained their characteristic spherical to ovoid shape, distinct refractive cytoplasm, and primary–secondary cell configurations (Figure 4A–F). No phenotypic degradation or loss of structural integrity was observed after a year of continuous subculturing, validating the robustness of the MEM-based culture system.

### 3.4. Viability of E. leei

**Figure 5:**
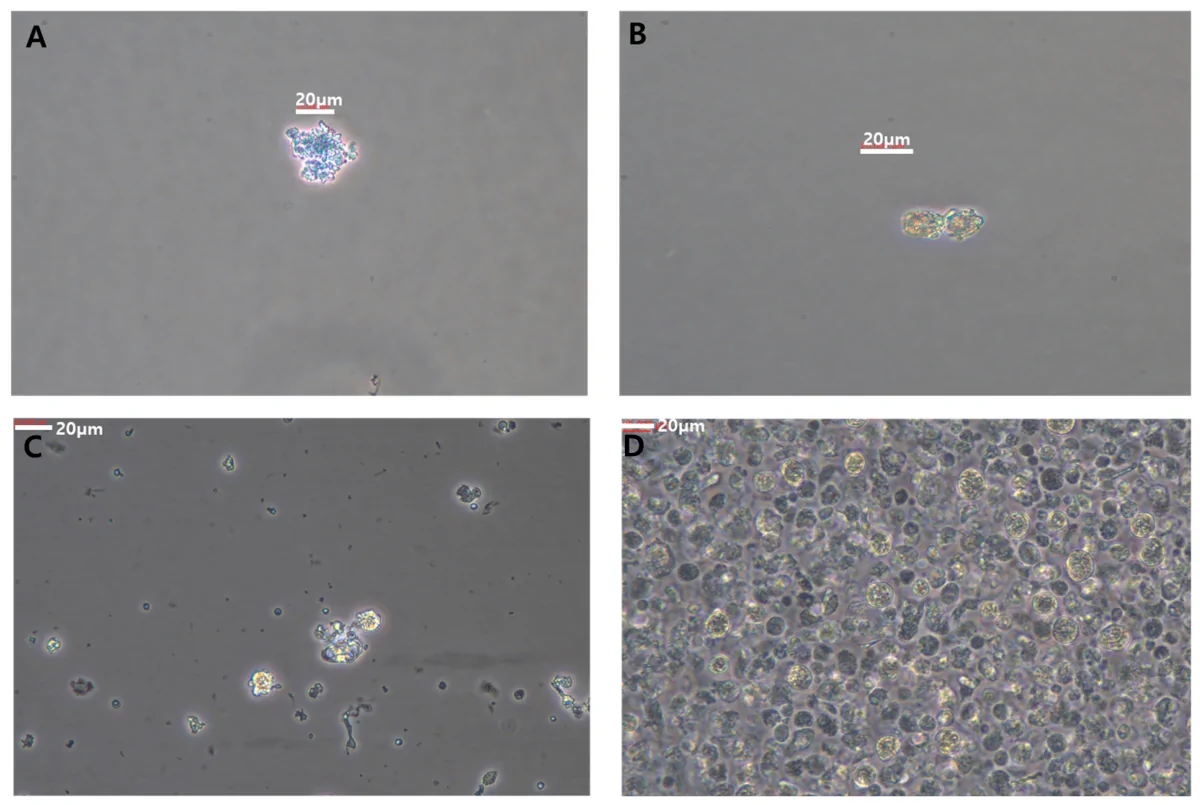
Viability assessment based on staining and morphological integrity. (**A**) Non-viable parasite (blue). (**B**) Viable parasite (unstained). (**C**) Dead parasite showing structural collapse. (**D**) Live parasite exhibiting clear, refractive cytoplasm. Scale bar = 20 μm.

The viability of the cultured parasites was assessed using Trypan blue exclusion and morphological criteria. Viable *E. leei* remained unstained with an intact, glistening cell membrane (Figure 5B,D), whereas non-viable individuals showed blue cytoplasmic infiltration and structural collapse (Figure 5A,C). Quantitative monitoring demonstrated that the highly enriched culture maintained a stable and high parasite viability rate throughout the 12-month period, with an annual mean viability of 82.4% ± 2.8%.

### 3.5. Optimization of In Vitro Proliferation Conditions

**Figure 6:**
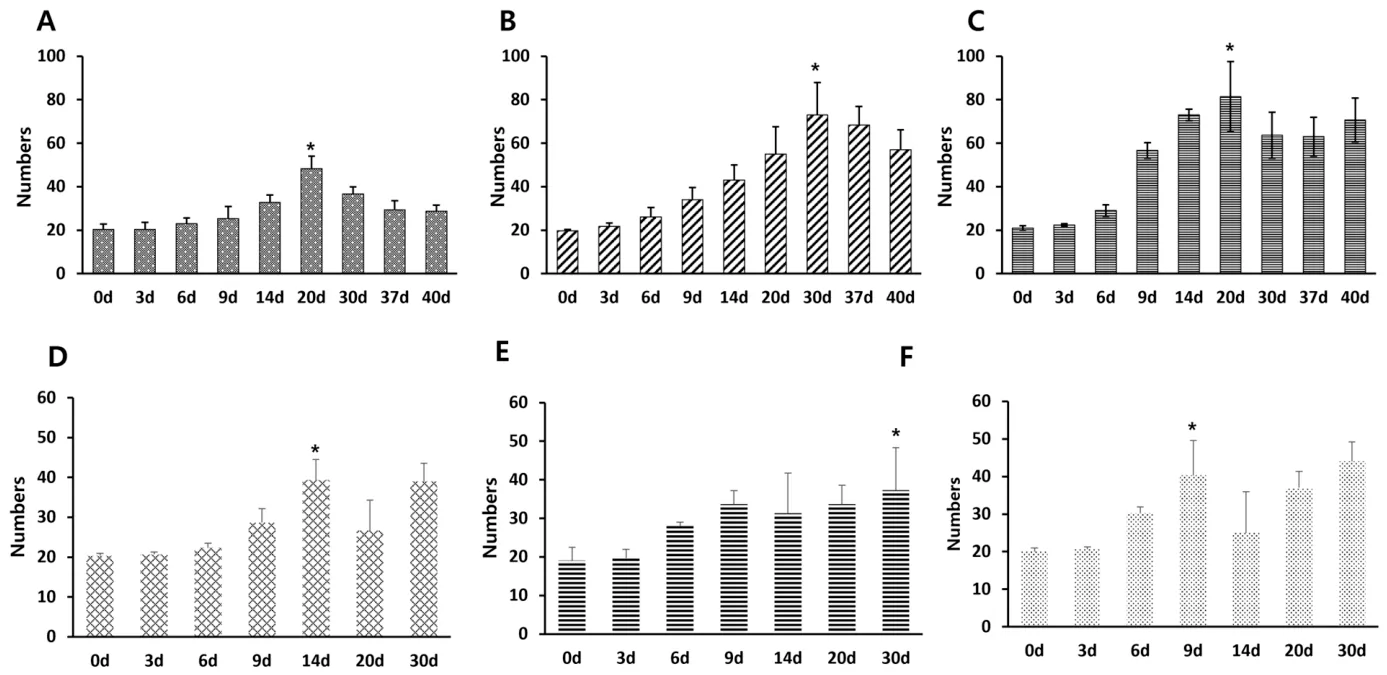
Proliferation dynamics of *E. leei* under varying temperatures and FBS concentrations. Parasites were cultured in MEM supplemented with 10% FBS at (**A**) 16 °C, (**B**) 20 °C, and (**C**) 25 °C. Parasites were cultured at a fixed temperature of 25 °C with varying FBS concentrations. (**D**) 10%, (**E**) 20%, and (**F**) 30%. Asterisks (*) denote statistical significance (*p* < 0.05) compared to day 0. Data are presented as mean ± SD (*n* = 3).

The proliferation rate of *E. leei* was quantitatively evaluated under varying environmental parameters to determine the optimal conditions for the in vitro system. Incubation temperature was found to have a measurable impact on growth dynamics. The parasites exhibited a stable doubling time of 20 days at both 16 °C and 25 °C. However, at 20 °C, the doubling time was extended to 30 days, indicating a reduction in the rate of replication at this specific temperature (Figure 6A–C). The concentration of FBS also significantly influenced the frequency of parasite replication. Under a fixed temperature of 25 °C, the shortest doubling time of 9 days was recorded in the medium supplemented with 30% FBS. In comparison, doubling times of 14 days and 30 days were observed at FBS concentrations of 10% and 20%, respectively (Figure 6D–F). In summary, the maximum proliferation rate in this study was recorded at an incubation temperature of 25 °C with 30% FBS supplementation.

### 3.6. Molecular Consistency and Phylogenetic Identity

**Figure 7:**
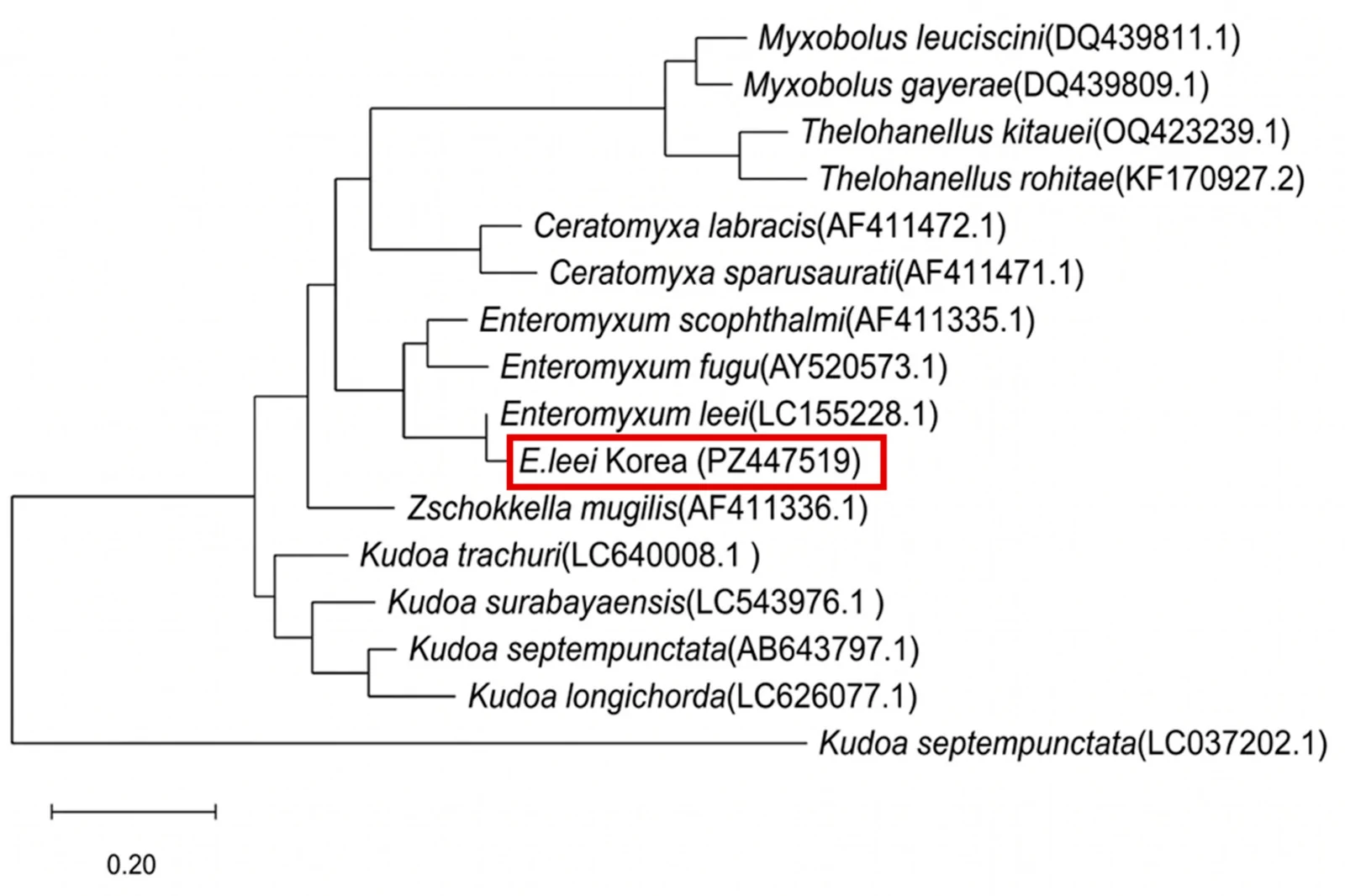
Phylogenetic analysis of *E. leei*. Phylogenetic relationships: A maximum likelihood tree based on *18S rRNA* gene sequences. The isolated strain in this study is designated as *E. leei* Korea (red box). The tree shows the taxonomic position of the isolate within the *E. leei* clade, distinct from other fish parasites such as *Kudoa* spp., *Thelohanellus* spp., and *Scuticociliates*. Robustness of the nodes was evaluated using 1000 bootstrap replicates. Detailed isolation hosts, countries, and years for all reference sequences are comprehensively summarized in Table S2.

The genetic identity of the cultured strain was monitored monthly. PCR amplification of the *18S rRNA* gene consistently yielded a specific 1468 bp band across all time points (1 to 12 months), indicating no loss of genetic material or contamination by other myxozoans (Figure S1A–E). Sequence alignment analysis revealed that the 1468 bp fragment of the *18S rRNA* gene shared high sequence homology, exhibiting a 99.8% to 100% nucleotide identity with homologous *E. leei* isolates previously deposited in GenBank (LC155258.1 from Republic of Korea). Phylogenetic analysis utilizing a 1412 bp alignment length confirmed that the isolate clustered definitively within the *E. leei* clade (Figure 7). This molecular evidence confirms that the long-term in vitro culture consists of a pure lineage of *E. leei*, maintaining high sequence homology with established reference strains while remaining phylogenetically distinct from other major aquatic parasitic groups.

### 3.7. Characterization of the In Vitro Proliferative Cycle

**Figure 8:**
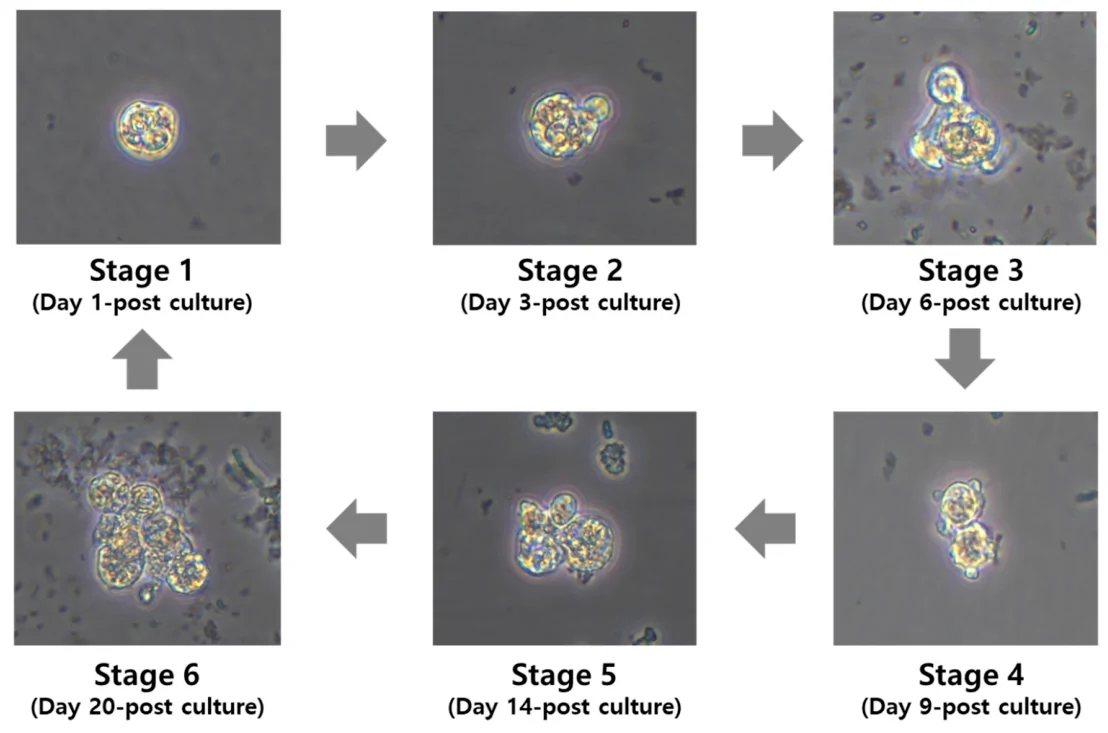
Sequential morphological development of *E. leei* proliferative stages during long-term in vitro culture. The 20-day proliferative cycle of *E. leei* observed in the established culture system progressed through six distinct developmental stages. Stage 1 (Day 1), initial trophozoite stage following isolation, characterized by a distinct cytoplasmic boundary and a primary nucleus. Stage 2–3 (Day 3–6), early proliferative phase showing internal budding; secondary cells are observed developing within the primary cell. Stage 4–5 (Day 9–14), intermediate expansion phase; the parasites exhibit multinucleation and increased cellular volume, forming early-stage clusters. Stage 6 (Day 20), mature cluster formation and initiation of the next proliferative cycle; multicellular aggregates are ready to release new proliferative stages, ensuring the continuity of the in vitro culture.

The in vitro development of *E. leei* followed a predictable 20-day cycle, progressing through six distinct stages. The cycle initiated with small, uni-nucleated trophozoites (stage 1) that underwent internal budding to form secondary and tertiary cells (stages 2–3). By day 14, these developed into intermediate multicellular aggregates (stages 4–5). The cycle culminated on day 20 (stage 6) with the formation of mature, complex clusters capable of releasing new daughter cells to initiate the subsequent round of asexual proliferation (Figure 8). This continuous cycle demonstrates that the in vitro system successfully mimics the proliferative phase observed in the host intestine.

## 4. Discussion

The establishment of a long-term in vitro culture system for *E. leei* represents a significant technical breakthrough, as this myxozoan has been widely regarded as an unculturable obligate parasite. While previous studies have predominantly relied on in vivo cohabitation or intubation models to maintain the parasite [7,21], these methods are limited by host immune interference, high maintenance costs, and the inability to observe real-time cellular dynamics. In this study, we successfully maintained *E. leei* isolates from *P. olivaceus* for over 12 months, achieving a stable proliferative cycle that mimics the physiological environment of the host intestine.

A key factor in this study was the implementation of the physical aggregation technique. Unlike conventional enzymatic digestion or filtration methods used for other protozoan parasites, which often damage the delicate primary cells of myxozoans [21,22], our approach minimized mechanical stress while effectively removing host-derived contaminants such as erythrocytes and epithelial debris. The qPCR data further validated this method, showing a 99.1% reduction in bacterial *16S rRNA*. This is a critical advancement compared to earlier attempts where rapid degradation of the culture medium occurred due to the proliferation of opportunistic bacteria and host cell lysis. By achieving high purity, we were able to sustain the asexual budding process, which is the primary mode of expansion for *E. leei* within the intestinal mucosa.

The 20-day proliferative cycle observed in our system provides the first detailed in vitro evidence of the sequential development of *E. leei*. The transition from unicellular trophozoites to complex, multinucleated plasmodia containing internal secondary cells closely aligns with the proliferative patterns previously identified in the intestinal epithelium of other flatfish [23]. While earlier studies primarily relied on in vivo sampling or short-term intestinal explants to describe these stages [23], our system demonstrates that the longitudinal asexual proliferative phase—from initial budding to the maturation of multicellular aggregates—can be fully stabilized and maintained in vitro for over 12 months, providing a robust continuous platform despite the co-aggregation of microscopic host cell remnants.

A notable observation in our in vitro system is the regularity of the 20-day cycle, which suggests programmed developmental kinetics independent of fluctuating host physiological signals. This finding extends the understanding of myxozoan biology, as previous in vivo observations were often confounded by the stochastic nature of parasite distribution and host immune responses [21]. By establishing stage 1 through stage 6 under controlled conditions, we have shown that the physical aggregation and optimized nutrient availability (30% FBS) effectively mimic the microenvironment of the *P. olivaceus* posterior intestine.

The transition from single trophozoites to multicellular aggregates observed in stage 6 closely mirrors the internal budding described in histological sections of infected *P. olivaceus* [8]. This morphological consistency through 12 months of subculturing proves that our culture conditions—specifically the 25 °C incubation and high FBS supplementation—provide the necessary biochemical cues for the parasite to bypass the need for continuous host signaling.

The technical challenge of cryopreservation remains a significant hurdle in aquatic parasitology. Similar to other major fish parasites like *Scuticociliates* (*Philasterides dicentrarchi*), *E. leei* appears to be highly sensitive to standard freezing protocols, often resulting in a total loss of viability upon thawing. It is noteworthy that for *P. dicentrarchi*, a relatively accessible ciliate, an optimized cryopreservation protocol was only recently established, highlighting that the stationary phase of growth is a critical factor for post-thaw survival [24]. Given that myxozoans possess a more complex multicellular structure, they are likely even more susceptible to osmotic shock and membrane rupture during freezing. Therefore, our success in achieving 12 months of continuous subculturing provides the most viable and reliable alternative for maintaining a live library of *E. leei* for experimental use.

Furthermore, the genetic stability confirmed by *18s rRNA* sequencing throughout the culture period ensures that the isolated strain, designated as *E. leei* Korea, remains taxonomically distinct from other co-infecting pathogens such as *Kudoa septempunctata*. This molecular validation is essential for establishing the highly enriched *E. leei* as a standardized experimental model.

The findings of this study provide a crucial link between in vitro observations and field-based clinical pathology. The optimal growth observed at 25 °C directly correlates with the peak incidence of emaciation disease in *P. olivaceus* farms during high-temperature seasons [3]. Moreover, the ability to maintain the parasite for 12 months without morphological drift suggests that the essential nutritional and biochemical requirements, which were previously thought to be host-dependent, can be satisfied through optimized in vitro conditions. While the lack of a successful cryopreservation protocol remains a challenge, as seen in other delicate aquatic parasites like *Scuticociliates*, our continuous subculturing method ensures a reliable and standardized source of *E. leei* for long-term experimental use.

## 5. Conclusions

This study established the first standardized in vitro culture system for *Enteromyxum leei*, achieving stable maintenance for over 12 months. By implementing a physical aggregation technique, we bypassed isolation challenges and achieved high-purity cultures with a 99.1% reduction in bacterial contamination. Our findings reveal that *E. leei* possesses an intrinsic 20-day asexual proliferative cycle that can proceed continuously under optimized in vitro nutrient conditions. Although further research is required to stimulate full sporogony and verify host infectivity, this standardized long-term subculturing method provides a highly enriched, robust live library for consecutive antiparasitic screening, bypassing a critical bottleneck in emaciation disease management.

## Figures and Tables

**Table 1:** Primers used for quantitative real-time PCR.

Primer	Target Species	Accession Number	Sequence (5′–3′)	Target Size (bp)	Reference
Po_β-actin_F	*Paralichthys olivaceus*	HQ386788.1	CTGGACTTCGAGCAGGAGATG	120	This study
Po_β-actin_R			GGCCTCTGGACAACGGAAC		
U16SRT-F	Variable bacteria	AB831972.1	ACTCCTACGGGAGGCAGCAGT	180	[18]
U16SRT-R			TATTACCGCGGCTGCTGGC		
*E. leei 18S rRNA*_F	*Enteromyxum leei*	KF056890.1	TGCTGCAGTTAAAAAGCTCGT	149	This study
*E. leei 18S rRNA*_R			GCACACTCCGCAAGTCACTA		

## Data Availability

The original contributions presented in this study are included in the article/Supplementary Materials. Further inquiries can be directed to the corresponding author.

## Supplementary Materials

The following supporting information can be downloaded at: https://www.mdpi.com/article/10.3390/pathogens15080836/s1, Table S1: qPCR results for purification efficiency validation; Table S2: GenBank accession numbers, host species, geographical origins, and isolation years of the myxozoan *18S rRNA* sequences used for phylogenetic analysis in this study; Figure S1: Molecular identification of *E. leei* during long-term in vitro maintenance. PCR amplification of the 18S rRNA gene using species-specific primers, consistently yielding a 1468 bp product at (A) 1 month, (B) 2 months, (C) 3 months, (D) 6 months, and (E) 12 months post-isolation. (M: molecular weight marker; PC: positive control).
