# Supplementary material for: Stable In Vitro Cultivation of the Myxozoan Enteromyxum leei for over 12 Months: Integration of a Physical Aggregation Technique for Long-Term Antiparasitic Interventions
Source: Pathogens. 2026 Aug 10;15(8):836. doi: 10.3390/pathogens15080836 (PMC13516221; doi:10.3390/pathogens15080836)
Supplement: Supplementary file 1 [file pathogens-15-00836-s001.zip › pathogens-4341656-supplementary.pdf]

Table S1. qPCR results for purification efficiency validation

| Target | Gene | Sample | Mean CT± SD | ΔCT | $2^{ΔΔCT}$ |
|---|---|---|---|---|---|
| *Enteromyxum leei* | *18s rRNA* | Pre | 21.27 | - | - |
| | | Post | 29.27 | - | - |
| Variable bacteria | *16s rRNA* | Pre | 27.15±0.15 | 5.88 | 1 |
| | | Post | 32.71±0.31 | 3.44 | 5.41 |
| *Paralichthys olivaceus* | *β-actin* | Pre | 12.17±0.27 | -9.1 | 1 |
| | | Post | 26.96±0.03 | -2.31 | 0.009 |

Table S2. GenBank accession numbers, host species, geographical origins, and isolation years of the myxozoan *18S rRNA* sequences used for phylogenetic analysis in this study

| | GenBank Number | Isolated host | Isolated year | Isolated country |
|---|---|---|---|---|
| *Myxobolus leuciscini* | DQ439811.1 | *Leuciscus cephalus* | 2008 | Hungary |
| *Myxobolus gayerae* | DQ439809.1 | *Leuciscus cephalus* | 2008 | Hungary |
| *Thelohanellus kitauei* | OQ423239.1 | *Cyprinus carpio* | 2023 | China |
| *Thelohanellus rohitae* | KF170927.2 | *ND* | 2013 | India |
| *Ceratomyxa labracis* | AF411472.1 | *Scophthalmus maximus* | 2002 | Spain |
| *Ceratomyxa sparusaurati* | AF411471.1 | *Scophthalmus maximus* | 2002 | Spain |
| *Enteromyxum scophthalmi* | AF411335.1 | *Scophthalmus maximus* | 2002 | Spain |
| *Enteromyxum fugu* | AY520573.1 | *Takifugu rubripes* | 2004 | Japan |
| *Enteromyxum leei* | LC155258.1 | *Paralichthys olivaceus* | 2016 | Korea |
| *Enteromyxum leei* | PZ447519 | *Paralichthys olivaceus* | 2026 | Korea |
| *Zschokkella mugilis* | AF411336.1 | *Scophthalmus maximus* | 2002 | Spain |
| *Kudoa trachuri* | LC640008.1 | *Caranx hippos* | 2022 | Japan |
| *Kudoa surabayaensis* | LC543976.1 | *Mugil cephalus* | 2021 | Japan |
| *Kudoa septempunctata* | AB643797.1 | *Paralichthys olivaceus* | 2011 | Japan |
| *Kudoa longichorda* | LC626077.1 | *Caranx hippos* | 2022 | Japan |
| *Kudoa septempunctata* | LC037202.1 | *Paralichthys olivaceus* | 2015 | Japan |

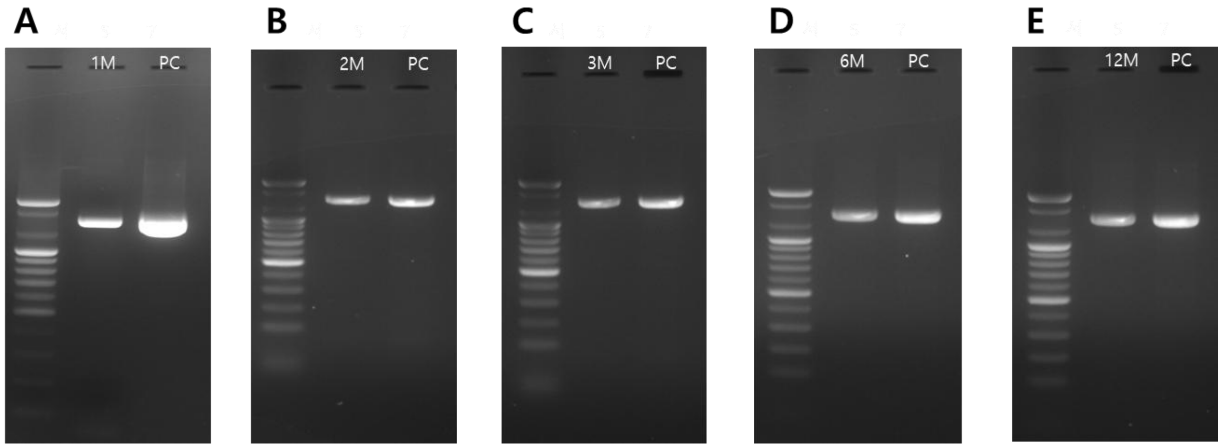


**Figure S1.** Molecular identification of *E. leei* during long-term in vitro maintenance. PCR amplification of the 18S rRNA gene using species-specific primers, consistently yielding a 1468 bp product at (A) 1 month, (B) 2 months, (C) 3 months, (D) 6 months, and (E) 12 months post-isolation. (M: molecular weight marker; PC: positive control).
